# Seminal plasma modulates post-thaw longevity and motility of frozen sperm in dromedary camel

**DOI:** 10.5713/ab.23.0136

**Published:** 2023-08-23

**Authors:** Fahimeh Seyedasgari, Behnam Asadi, Ellen Kim

**Affiliations:** 1 Camel Advanced Reproductive Center, Zabeel Office, Government of Dubai, Dubai 5928, United Arab Emirates

**Keywords:** Insemination, Pregnancy, Seminal Plasma

## Abstract

**Objective:**

This study investigated the effect of adding seminal plasma to frozen-thawed semen on the quality of sperm and pregnancy following insemination in dromedary camels.

**Methods:**

In experiment 1, the frozen-thawed semen from 9 collections (3 bulls) was further diluted with either the base extender or homologous seminal plasma (HSP). In the second experiment, a pooled sample of frozen-thawed semen was diluted with either seminal plasma from another three bulls. Live percentage, total and progressive motility, functional and acrosome integrity, and sperm kinematics were evaluated at 15, 60, and 120 minutes post-thawing and compared to the non-treated control. In experiment 3, frozen semen was used to inseminate camels in the following experimental groups: 1-Single insemination with double dose undiluted frozen semen (n = 9); 2-Re-insemination in 6 hours with undiluted semen (n = 13); 3-Single insemination with HSP treated sperm (n = 14).

**Results:**

Frozen-thawed sperm diluted in HSP or the non-homologous seminal plasma from Bull C indicated an improvement in all parameters after 1 hour post-thawing incubation (p<0.05). The proportion of total and progressively motile sperm did not drop significantly at 60 minutes post-thawing when diluted with the seminal plasma of Bull C (p>0.05). Double insemination with nontreated sperm and single insemination with HSP-treated sperm resulted in similar pregnancy rates (15.3% vs 21.4%, p>0.05). None of the camels conceived with double-dose single insemination of nontreated sperm.

**Conclusion:**

Seminal plasma improves sperm longevity and motility after thawing in dromedary camel with a significant between-bull variation in effect. Low post-thaw sperm longevity might be the cause behind the low pregnancy rates in frozen semen insemination of dromedary camels.

## INTRODUCTION

Seminal plasma (SP) forms the acellular fraction of the ejaculate, which comprises an array of components produced mainly by the accessory sexual glands and the testicles and epididymis. It is classically considered a vehicle and nutrient source for sperm, but evidence indicates the crucial involvement of its components in physiological pathways related to sperm function, such as capacitation [1], chromatin stability [2], interaction with oviductal epithelia to form sperm reservoir [3], and signaling the immune system of the uterus and cervix [4]. Interestingly, evidence is gathering that the SP also impacts the outcome of fertility by modulating the maternal environment in preparation for a healthy pregnancy [5] and by directly affecting the offspring’s metabolic phenotype [6] and performance [7] through modulating sperm longevity.

The SP is generally made up of energy sources for sperm and a variety of ions, amino acids and peptides, low and high-molecular-weight proteins, lipids, hormones, enzymes, cytokines, and antioxidants [8]. The rheological properties of the ejaculate in several species, such as humans, equine, porcine, and camelids, are attributed to the presence of specific components in SP [9]. The proteomic studies on humans have identified Seminogelin B as the responsible factor behind post-ejaculation jellification of semen [10], while a comparative study between high and low viscous camelid semen samples, found the protein fraction of the SP, including Mucin B to be more abundant in the former group [11].

Under the context of sperm preservation, despite some controversy, there is a general consensus on the deleterious effects of SP on the efficiency of sperm freezing [12,13], and regardless of the cause, hyper-viscosity in some samples has been shown to impose additional hurdle for sperm preservation in humans [14]. In the case of camelids, the intrinsic viscous nature of the whole ejaculate is attributed to SP and accounts for a significant part of the difficulties in sperm handling and processing [15]; therefore, elimination of viscosity as a prerequisite for downstream procedures such as successful freezing has been extensively described in these species [16]. To the best of our knowledge no study has ever evaluated the possible modulatory effects of SP on sperm function and fertility in camels. In this regard, studies on other species had reported improved quality of chilled and frozen-thawed cryopreserved sperm when samples were exposed to SP after thawing [17–19], resulting in improved pregnancy rates after insemination [20–22].

This study is a preliminary look into the effects of SP on frozen-thawed semen in dromedary camels. In light of previous reports in other species, we assumed that SP should serve physiological functions that modulate sperm characteristics in favor of a higher chance for fertilization. In an attempt to improve the pregnancy after insemination with frozen-thawed semen, the success rate of which is far from efficient in camelids [23,24], we hypothesized that the possible evolutionary role of SP as a carrier for sperm *in-vivo* could be exploited for improving the outcome of frozen semen insemination in dromedary camel.

## MATERIALS AND METHODS

### Experimental animals and location

All experimental procedures were internally reviewed for compliance with to ARRIVE code of conduct and the UAE regulations on animal care and use. The study took place at Camel Advanced Reproductive Technologies Center, Dubai, United Arab Emirates, during the breeding season of 2020. Six individually caged dromedary bulls of local breeds with a proven record of fertility, aged 8 to 12 years, were used for semen collection from December to April. Female camels were procured from the local farms and admitted to the herd after passing a screening test for brucellosis, trypanosomiasis, and the evaluation of breeding soundness. Daily, bulls were fed a 10 kg mixture of Sudan grass, alfalfa hay, and clover, and 2 to 3 kg of concentrate formulated for racing bulls per capita. For females, the ratio consisted of a mixture of 10 kg alfalfa hay and Sudan grass per/head/day. All animals had *ad libitum* access to drinking water.

### Experimental design

Three series of prospective interventional experiments were carried out to investigate the effect of SP on the post-thaw quality of frozen semen in dromedary camel. The ejaculates from the first three bulls were processed to separate SP by centrifugation (n = 9) or were subjected to freezing (n = 9). Experiment 1 aimed to evaluate the effect of adding homologous SP on the post-thaw quality of sperm. In each experimental block, four straws from each collection/freezing were thawed, and the contents were pooled before equal allocation to either of the three experimental groups with the following further dilutions (1:1 v/v): 1- Control (non-diluted); 2-Base medium; 3-Seminal plasma from the same bull (HSP). Samples were examined for viability, total motility, functional and acrosome integrity, and sperm kinematics at 10-, 60-, and 120-minute of incubation at 37°C post-thawing. The second experiment aimed to evaluate the possible between-bull variations among the seminal plasma with regard to their effect on sperm quality. For this experiment, frozen samples from all freezing sessions (one straw each) were pooled after thawing and randomly diluted (1:1 v/v) with the non-HSP from another three bulls in three replicates. The respective sperm-related parameters were evaluated at 10-, 60-, and 120-minutes post-thawing. Experiment 3 investigated whether the addition of HSP to the frozen-thawed sperm would improve pregnancy rates after insemination. We hypothesized that the improved longevity of sperm in the treatment group of experiment 1 would improve the chances of pregnancy with frozen semen. For this purpose, 39 camels were randomly inseminated using frozen-thawed sperm in the following arrangement regarding the time after induction of ovulation: 1-Control group: Single insemination with double dose at 26 hours (n = 9); 2-Double insemination with a single dose at 26 and 32 hours (n = 15); and 3-Single insemination with single dose+ HSP, at 26 hours (n = 15). A total of 39 and 14 collections from 3 bulls were used for obtaining frozen samples and seminal plasma for this experiment, respectively.

### Semen collection

A bovine artificial vagina was used for semen collection into a water-jacketed glass tube with an inner temperature of ~40°C, as described elsewhere [16]. Collected semen was immediately transferred to the laboratory for initial evaluation. Samples with less than 2.5 mL in volume, dirt-polluted samples, and those with mass vibration of less than 75% were discarded.

### Semen processing and preservation

Unless otherwise stated, all chemicals were procured from Sigma (Sigma- Aldrich Co, St Louis, MO, USA). The base medium for the dilution of samples in all experiments was Green Buffer (G.B.; IMV, L′ Aigle, France) and 20% egg yolk plasma (v/v), containing 6 mM of N-acetyl cysteine for centrifugation purposes in the freezing groups. The plasma egg yolk was prepared by two times centrifugation of PBS-diluted yolk content (1:1, v/v) of fresh commercial eggs at 10,000×g for 45 minutes [25]. After the initial dilution of semen (1:1, v/v) and gentle pipetting for 2 minutes, semen was kept at room temperature for 15 minutes before centrifugation at 800 g for 10 minutes. The centrifugation tube contained 1 mL of a dense liquid cushion (Cushion Fluid, Minintube, Germany) in the bottom. For the preparation of seminal plasma, the supernatant was harvested and subjected to another round of centrifugation at 1,200 g for 15 minutes to remove the cell debris and was kept frozen at −80°C until further use. The final solution contained ~ 50% SP. In the freezing groups, pellet was resuspended in the base medium, and the concentration was adjusted to 400×10^6^ sperm/mL. Semen was cooled to 5°C within 2 hours, and diluted with the freezing extender (6% glycerol; 1:1 v/v). The filled straws were subjected to freezing by 15 minutes of exposure to liquid Nitrogen vapor from a distance of 4 cm above the surface in a modified Styrofoam box before being plunged into the bottom of the container.

### Experimental treatments

Frozen seminal plasma was placed in a water bath prior to the thawing of sperm to reach 37°C. Frozen straws were thawed by exposure to hot water (40°C for 40 seconds) and immediately subjected to further dilution with HSP or base medium in experiment 1 (1:1 v/v). For experiment 2, samples were pooled and diluted with the seminal plasmas from another three bulls (1:1 v/v). For inseminations, the inseminate dose was adjusted to 200×10^6^ progressively motile frozen-thawed sperm before randomly assigning it to either the control or HSP groups.

### Sperm assessments

#### Viability, motility, and acrosome integrity

Total motile sperms were counted by observing 200 sperms on a wet smear (10 μL) under a phase contrast microscope equipped with a warm stage (Model: BX53; Olympus, Tokyo, Japan). Progressive motility refers to the forward-moving sperm at slow, medium, and fast paces, excluding oscillating sperm. The viability of samples was assessed by Eosin B-fast green staining as described elsewhere [26]. Briefly, 10 μL of diluted sample was mixed with 10 μL of eosin B-fast green, and the dried smear was examined under 100× magnification by an experienced operator, recording the number of cells with non-permeable membrane and intact acrosomes out of 200 counted cells.

#### Functional integrity

The hypo-osmotic swelling test was carried out as described elsewhere [27]. In brief, 100 μL of the post-thaw sample was added to 1 mL of 1.25% Fructose solution and incubated for 45 min at 37°C. The proportion of sperm cells with coiled/swollen tails out of 200 observed cells was reported.

#### Kinematics

The measured sperm kinematics included curvilinear velocity (VCL, μm/s), average path velocity (VAP, μm/s), straight-line velocity (VSL, μm/s), mean amplitude of lateral head displacement (ALH, μm), and beat cross frequency (BCF, Hz). Kinematics were measured by means of a computer-assisted sperm analysis (CASA; ISAS v1, Proiser R+D S.L., Paterna, Spain) as described elsewhere [28]. Briefly, 4 μL sample was loaded on the special chamber (20 μ, Sperm Track, Proiser, Spain), and 100 consecutive digitalized images within a time-lapse of 2 seconds were captured in a minimum of 5 fields to calculate the respective parameters by the software.

### Camel preparation and inseminations

A cohort of camels aging between 7–10 years with no gross reproductive complication and a negative uterine culture as described elsewhere [29] were considered for inseminations and randomly assigned to either of the experimental groups. Upon planned insemination, camels with a single dominant follicle sizing 12 to 15 mm received an intravenous injection of 20 μg of Buserelin acetate (Gestar, Over, Santa Fe, Argentina) with the insemination planned 26 to 32 hours later. For insemination, a 70 cm covered A.I. catheter (Equivet, Langeskov, Denmark) was introduced into the cervix and guided towards the uterine horn by rectal manipulation. The inseminate was delivered in the uterine horn ipsilateral to the dominant follicle. Camels that did not ovulate until 32 hours post-induction were excluded from the rest of the experiment to avoid the confounding effect of ovulation timing on the results. Pregnancy was checked 23 days post-insemination based on the presence of the embryo proper or the embryonic heartbeat along a viable corpus luteum.

### Statistical analysis

A mixed-model repeated measures analysis compared the effect of experimental treatments with treatment and time included in the model as the fixed factors and the ejaculate nested within the bull as the random factor. Values were compared by least significant difference correction and presented as mean±standard error of the mean. The pregnancy rates were compared between groups by Fisher’s exact extension of the Chi-square test of independence. All statistical procedures were performed by the SPSS software package v20.0.

## RESULTS

### Experiment 1

**Figure 1:**
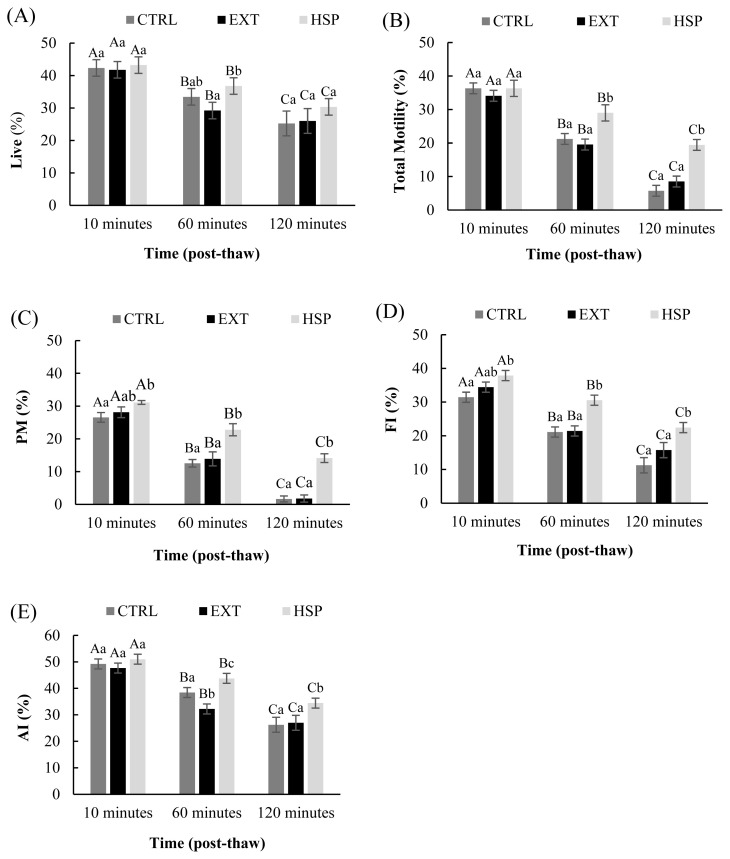
(A)–(E) Sperm quality in control (CTRL), extender (EXT), and homologous seminal plasma (HSP) evaluated at 10, 60, and 120 minutes after thawing. ^A–C a–c^ Different capital and small letters indicate significant differences in time and among treatments, respectively (p<0.05). (A) Live percentage (time effect: CTRL p<0.001; EXT p<0.007, HSP p<0.010; treatments: 10 minutes p<0.533, 60 minutes p<0.010, 120 minutes p<0.007); (B) Total motility (time effect: CTRL p<0.000; EXT p<0.000, HSP p<0.000; treatments: 10 minutes p<0.312, 60 minutes p<0.000, 120 minutes p<0.000); (C) progressive motility (time effect: CTRL p<0.000; EXT p<0.000, HSP p<0.000; treatments: 10 minutes p<0.012, 60 minutes p<0.000, 120 minutes p<0.000); (D) Functional integrity (time effect: CTRL p<0.000; EXT p<0.001, HSP p<0.001; treatments: 10 minutes p<0.005, 60 minutes p<0.000, 120 minutes p<0.001); (E) Acrosome integrity (time effect: CTRL p<0.000; EXT p<0.002, HSP p<0.002; treatments: 10 minutes p<0.192, 60 minutes p<0.016, 120 minutes p<0.011).

All sperm-related parameters were exacerbated with time in all experimental groups (p<0.01; Figure 1A–E). Adding HSP to the frozen-thawed sperm improved the proportion of total motile and acrosome intact sperms at 1- and 2-hours post-treatment, compared to the control and extender groups (p<0.05; Figure 1A, B, and E). Progressive motility and functional integrity of sperm were higher in the seminal plasma-treated group 10 minutes after thawing compared to the control (31.11±0.58 vs 26.55±1.47, and 37.89±1.51 vs 31.44± 1.51) with a carry-over effect until the end (p<0.05; Figure 1C, D), while simple addition of the extender to the frozen-thawed semen did not improve any of the respective parameters at any time point compared to the control or HSP group (p> 0.05, Figure 1A–E).

Table 1 compares the changes in sperm kinematics among control, extended, and seminal plasma-treated groups over the incubation time post-thawing. The velocity of frozen-thawed samples, as well as values for ALH and BCF, did not drop significantly between thawing and 60 minutes when sperm was diluted in extender or HSP, with the latter treatment showing a carry-over effect until 120 minutes (p>0.05; Table 1). VCL, VSL, and BCF were similar between control and HSP (p>0.05), while VAP was highest in the extender group (p<0.5) at 10 minutes after thawing (Table 1). The velocity of sperm was the lowest in control at 60 and 120 minutes (p<0.05), while the respective values were similar between diluted groups at 60 minutes post thawing (p>0.05) but higher in HSP-treated group at 120 minutes (p<0.05; Table 1).

### Experiment 2

**Figure 2:**
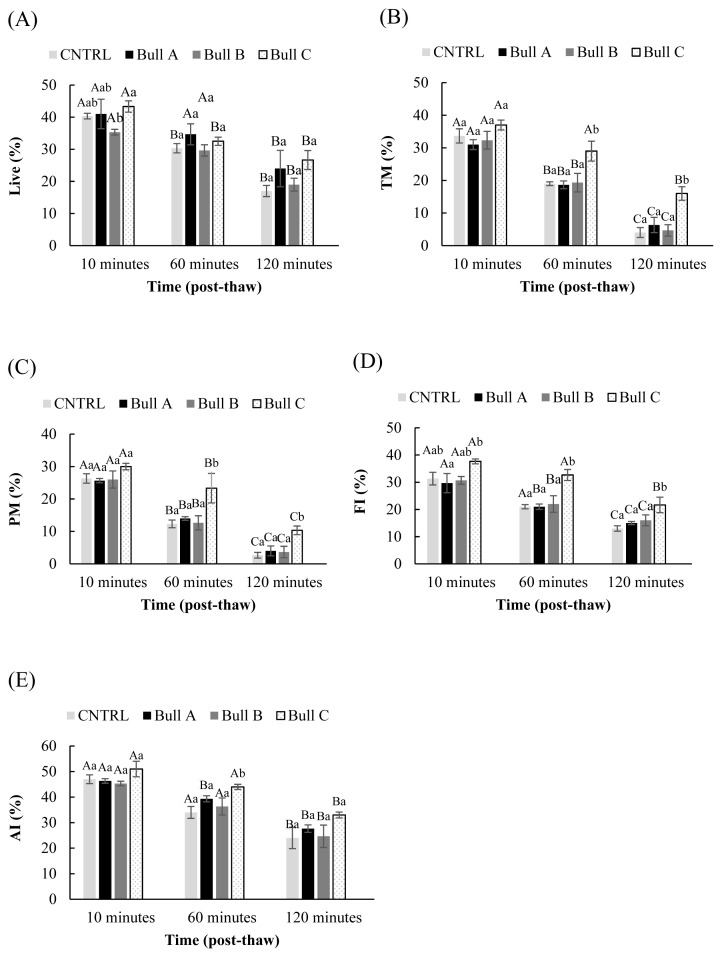
(A)–(E) Sperm quality among frozen-thawed semen diluted in seminal plasma from different bulls (A, B, and C) evaluated at 10, 60, and 120 minutes after thawing. ^A–C a–c^ Different capital and small letters indicate significant differences in time and among treatments, respectively (p<0.05). (A) Live percentage (time effect: CTRL p<0.009, Bull A p<0.023, Bull B p<0.023; Bull C p<0.021; treatments: 10 minutes p<0.037, 60 minutes p<0.135, 120 minutes p<0.084); (B) Total motility (time effect: CTRL P p<0.000, Bull A p<0.001; Bull B p<0.001, BULL C p<0.017; treatments: 10 minutes p<0.073, 60 minutes p<0.014, 120 minutes p<0.008); (C) Progressive motility (time effect: CTRL p<0.004, Bull A p<0.001; Bull B p<0.007, BULL C p<0.0.037; treatments: 10 minutes p<0.096, 60 minutes p<0.025, 120 minutes p<0.011); (D) Functional integrity (time effect: CTRL p<0.008, Bull A p<0.0038; Bull B p<0.038, BULL C p<0.001; treatments: 10 minutes p<0.038, 60 minutes p<0.004, 120 minutes p<0.059); (E) Acrosome integrity (time effect: CRTL p<0.005, Bull A p<0.000; Bull B p<0.000, BULL C p<0.024; treatments: 10 minutes p<0.064, 60 minutes p<0.005, 120 minutes p<0.079).

Figure 2A–E summarizes the changes in sperm-related parameters in pooled samples diluted in seminal plasma of different bulls as evaluated at 10-, 60-, and 120-minutes post-thawing. There was a significant between bull variation (p<0.000) and a progressive decline in all parameters between 10 minutes and 120 minutes post-thawing (p<0.000). Nevertheless, the proportion of total motile sperm as well as functional and acrosome intact cells did not drop significantly between 10 minutes (and 60 minutes (29.00±3.05, post thawing, when sperm was treated with seminal plasma from bull C (p>0.05; Figure 2B, C). The same group had the highest values for total motility, progressive motility, and functional integrity at 60 and 120 minutes (p<0.05; Figure 2D) while showing a trend for a significant difference in the proportion of acrosome intact cells throughout the post-thawing incubation, as compared to the other groups (p<0.064, <0.005, and <0.079 at 10, 60, and 120 minutes, respectively; Figure 2B).

### Experiment 3

**Figure 3:**
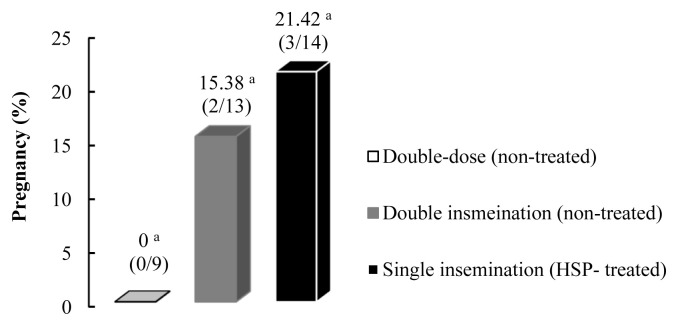
Comparative pregnancy rates after insemination with frozen-thawed sperm between single insemination with double dose non-treated semen, double insemination with non-diluted semen and single insemination with sperm treated with homologous seminal plasma. ^a^ Different letters indicate significant difference by Fisher’s exact test (p = 0.608).

A total of 36 camels that ovulated between 26–32 hours after induction were inseminated, excluding one animal in each of the double-dose single insemination and HSP groups and two camels in the double insemination group due to late ovulation. All inseminations were carried out within 10 minutes after the thawing of the sperm. Single insemination with a double dose of frozen-thawed semen resulted in no conception (0/9), and no further inseminations were carried out in this group. A total of 5 pregnancies were obtained with insignificant differences between the double insemination with frozen-thawed sperm (2/13) and frozen-thawed sperm diluted in HSP (3/14) (14.28% and 21.42%, respectively, p>0.05; Figure 3).

## DISCUSSION

The current study reports the effect of seminal plasma on frozen dromedary spermatozoa after thawing for the first time. Camelid seminal plasma has often been considered a hurdle to overcome due to the inherent hyper viscosity which results in the entrapment of sperm and prevents homogenous exposure of cells to cryoprotectants [15], which in turn, complicates the process of sperm freezing. The obtained results provide preliminary insight into the significant effect of seminal plasma on sperm longevity in camels. The findings are two fold: 1-Adding seminal plasma immediately after thawing of semen modulated the motility and functionality of sperm during *in-vitro* incubation, resulting in marked improvement of longevity. 2-The effect of seminal plasma shows between-subject variations among different bulls. Moreover, the current results provide indirect evidence that the longevity of sperm might be the primary determinant of the outcome in the frozen semen insemination of camels.

Results confirmed the modulatory effects of seminal plasma on the quality of frozen-thawed semen in dromedary camels (Figure 1A–E; Table 1). The effects were most evident at 60- and 120 minutes post-thawing when the motile proportion and kinematics of sperm and functional and acrosome integrity were highest in the HSP group. Similar observations were reported from other species. Exposure of sperm to whole seminal plasma has been shown to improve the motility pattern and acrosome intactness in ram resulting in compensation for the functional impairment observed in frozen sperm, which renders them inefficient in passing the cervix [20]. Post-thaw treatment of sperm in 50% autologous seminal plasma markedly improved total viability and motility of boar sperm during 4 hours of *in-vitro* incubation in sows, as compared to non-treated or 10% SP [19]. However, in the stallion, only a slightly higher acrosome and plasma integrity was observed in homologs (34.2%) and autologous (34.6%) SP groups compared to the control (31.4%; p<0.05) with no effect on viability and motility [30].

The well-documented modulatory effects of seminal plasma on sperm have been attributed to the components of this biological fluid, particularly its proteome [31]. Heparin-binding Spermadhesins have been associated with plasma membrane stabilization prior to capacitation [9] as well as the proportion of forward progressive sperm [32], while non-heparin-binding seminal proteins PSP-I and PSP-II displayed the ability to maintain high viability, motility, and mitochondrial activity of sperm *in-vitro* and the treatment has been suggested to improve the longevity of susceptible sperm during the process of sex-sorting [33]. The components of camelid seminal plasma have been briefly addressed in a previous report [11]; however, the main focus of the study has been to identify the cause behind the conserved hyper-viscosity of semen in these species. The current results indicate that further analysis of seminal plasma in camelids deserves attention for the improvement of insemination outcomes. Interestingly, a dose-dependent positive relationship between heparin-binding seminal proteins pre-freeze and post-thaw progressive motility of sperm indicates the possible beneficial effect of an isolated fraction of seminal plasma on the freezing process as well [32].

Variations in the type of seminal plasma proteins among different species are attributed to the differences in their origin and the sequence in which they are added to the ejaculate [9]. Still, within species, variations in the composition and effect of SP have also been documented [18,32,34,35]. Although treatment with HSP improved motility, and functional and acrosome integrity of frozen-thawed sperm (Figure 1; Table 1), the sample from only one bull had similar effects when the source of sperm was non-homologous to the SP (Figure 2A–E). In equine, there was a trend in the overall variation among the effect of seminal plasma from different stallions on motility (p<0.069), with the interaction between the origin of sperm and the origin of SP affecting the progressive motility during the chilled storage (p<0.05) [34]. The same study reported higher motility with homologous compared to heterologous SP but lower DNA integrity in either of the treatments compared to the control, except for one stallion that had less DNA damage in HSP [34]. In another study, the pregnancy rates in inseminated sows were increased from 9% to 70% when frozen sperm from poor freezer boars was thawed in a solution containing 10% SP of high fertility boars, with the treatment shown to improve acrosome intactness of sperm and suppress the expression of 15 kDa phosphoproteins [21]. These variations in SPs within groups of animals in the same species, are probably due to large differences in the protein content between the good and poor freezers, including a higher concentration of antioxidants and aminopeptidase N in the former group [35].

Conception under the frozen sperm insemination program in camelids is still far from efficient compared to other farm animals, in which average pregnancy rates of >50% are not uncommon. In the current study, no pregnancy was obtained when 400 million progressively motile sperms were given at a single dose, but inseminating camels with the same number of motile sperms split between two inseminations with a 6-hour gap in between increased the pregnancy rates to 15.38% (2/13; Figure 3). On the other hand, single insemination with 200 million progressively motile sperm exposed to HSP resulted in 3 pregnancies out of 14 inseminations (21.4%), with an insignificant difference between the two latter groups (p>0.05; Figure 3). In the very few scientific insemination trials with frozen semen in Camelids, pregnancy rates of 7.9% and 26% have been achieved in lamas and alpacas, respectively [23,36]. In dromedary camels, improvement of post-thaw motility and kinematics of catalase-treated sperm (500 U/mL) was not accompanied by an improvement in pregnancy compared to a control group when camels were inseminated with pooled samples of 6 different bulls, achieving a total of 2 pregnancies out of 20 inseminations (10%) [24].

The multitude of factors involved in the success of frozen semen insemination makes the interpretation of the obtained results a difficult task. Breeding healthy mares as close to ovulation as possible has been shown to improve the results of frozen semen insemination [37], and therefore, camels ovulating before 26 hours were not inseminated and those ovulating after 32 hours post-induction were not included in the study to minimize the impact of ovulation timing on the results. Achieved pregnancies in the HSP-treated group provide a clue that a minimum of 200 million progressively motile frozen-thawed sperm should suffice for the establishment of pregnancy, and the longevity of frozen-thawed sperm probably is the primary determinant of the outcome. In line with this hypothesis, dilution of frozen-thawed boar semen in 50% autologous seminal plasma resulted in higher viability and motility of frozen sperm with a subsequent rise in the pregnancy from 64% to 80%, showing no insignificant difference compared to the fresh insemination (84%) [19]. In a trial on deep-horn insemination of donkeys, pregnancy was higher when frozen-thawed sperm was re-extended in a pooled sample of SP than INRA96 immediately before insemination (60.0% vs 22.2%; p<0.05) [22].

Alternatively, the establishment of pregnancy in the double insemination group might highlight the significance of the timing for performing the insemination. Nevertheless, single insemination with non-treated semen at 32 hours, did not result in any pregnancy in a follow-up trial (data not shown). The longevity of frozen-thawed dromedary sperm is very low under normal conditions (Figure 1), with half of the samples in the control group of the current study approaching viability values close to zero at 120 minutes post-thaw. Therefore, it can be hypothesized that such low longevity narrows the chances of *in-vivo* fertilization, but prolonging the availability of quality sperm by re-insemination or treatment with HSP, shown to improve longevity (Figure 1; Table 1), would eventually facilitate conception. Noteworthy, seminal plasma can modulate the proteome of the uterus in favor of higher chances for implantation [38]. However, when seminal plasma was infused into the uterus after insemination, no further improvement in pregnancy rate was obtained in cattle and horses through these mechanisms [39,40]. Much further work is required to clarify the effect of seminal plasma on dromedary sperm and the mechanisms through which these effects are exerted and orchestrated.

## CONCLUSION

In conclusion, seminal plasma of dromedary camel, which has often been studied for its deleterious effects on sperm processing in camelids, can modulate the longevity of frozen-thawed sperm and improve pregnancy rates with frozen semen inseminations, with marked between-bull variations in the effects. These preliminary insights are promising, and further research to identify which components of seminal plasma are involved in such modulations can possibly open the door for improvement in the semen technologies of the dromedary camel.

## Figures and Tables

**Table 1:** Kinematic values (mean±standard error of the mean) in frozen-thawed semen in control extender or homologous seminal plasma (HSP), during 120 minutes of incubation

Parameter	Treatment	Time			

		10 minutes	60 minutes	120 minutes	p-value
VCL (μm/s)	Control	63.89±2.92^A,a^	44.28±5.91^B,a^	5.70±5.70^C,a^	<0.000
	+Extender	64.82±2.22^A,a^	64.78±3.62^A,b^	26.45±10.66^B,b^	<0.000
	+HSP	69.28±3.49^A,a^	66.51±4.45^A,b^	58.91±3.94^A,c^	<0.096
	p-value	<0.026	<0.001	<0.020	
VAP (μm/s)	Control	25.06±0.63^A,a^	17.82±2.33^B,a^	2.20±2.20^C,a^	<0.000
	+ Extender	27.12±0.67^A,b^	25.86±1.27^A,a^	10.24±4.07^B,b^	<0.000
	+HSP	27.90±0.74^A,b^	26.16±1.07^A,b^	23.24±1.12^A,c^	<0.069
	p-value	<0.001	<0.001	<0.028	
VSL (μm/s)	Control	17.37±0.17^A,a^	11.31±1.66^B,a^	1.26±1.26^C,a^	<0.000
	+ Extender	21.84±0.67^A,b^	20.56±1.38^A,b^	7.78±3.10^B,b^	<0.000
	+HSP	18.62±0.42^A,a^	18.85±0.82^A,b^	16.74±0.99^A,c^	<0.372
	p-value	<0.000	<0.001	<0.032	
ALH (μm)	Control	2.91±0.10^A,a^	2.19±0.28^B,a^	0.28±0.28^C,a^	<0.001
	+ Extender	2.96±0.08^A,a,b^	2.95±0.13^A,b^	1.21±0.48^B,b^	<0.000
	+HSP	3.13±0.15^A,b^	3.07±0.15^A,b^	2.72±0.15^A,c^	<0.170
	p-value	<0.022	<0.002	<0.0032	
BCF (Hz)	Control	5.55±0.08^A,a^	4.58±0.65^B,a^	0.68±0.68^C,a^	<0.027
	+ Extender	6.44±0.17^A,b^	6.31±0.21^A,b^	2.44±0.98^B,a^	<0.000
	+HSP	5.83±0.17^A,a^	5.60±0.13^A,a^	5.56±0.32^A,b^	<0.578
	p-value	<0.000	<0.049	<0.005	

VCL, curvilinear velocity; VAP, average path velocity; VSL, straight-line velocity; ALH, amplitude of lateral head displacement; BCF, beat cross frequency.

A–C, a–c Different capital and small superscripts indicate significant differences in time and between treatments, respectively (p<0.05).
